# Microbiome-Independent Effects of Antibiotics in a Murine Model of Nosocomial Infections

**DOI:** 10.1128/mbio.01240-22

**Published:** 2022-05-25

**Authors:** Keenan A. Lacey, Sandra Gonzalez, Frank Yeung, Gregory Putzel, Magdalena Podkowik, Alejandro Pironti, Bo Shopsin, Ken Cadwell, Victor J. Torres

**Affiliations:** a Department of Microbiology, New York University Grossman School of Medicine, New York, New York, USA; b Kimmel Center for Biology and Medicine at the Skirball Institute, New York University Grossman School of Medicine, New York, New York, USA; c Department of Medicine, Division of Infectious Diseases, New York University Grossman School of Medicine, New York, New York, USA; d Antimicrobial-Resistant Pathogens Program, New York University Grossman School of Medicine, New York, New York, USA; e Division of Gastroenterology and Hepatology, Department of Medicine, New York University Grossman School of Medicine, New York, New York, USA; University of Rochester

**Keywords:** MRSA, antibiotics, pneumonia, antimicrobial resistance, murine model, AMR, ESKAPE, infection, mouse, murine models, pathogenesis

## Abstract

Methicillin-resistant Staphylococcus aureus (MRSA) is one of the most common causes of hospital-acquired pneumonia. To better manage patients with MRSA pneumonia, we require a greater understanding of the host-pathogen interactions during infection. MRSA research focuses on highly virulent and cytotoxic strains, which demonstrate robust phenotypes in animal models of infection. However, nosocomial infections are often caused by hospital-acquired MRSA (HA-MRSA) isolates that exhibit low cytotoxicity and few or no phenotypes in mice, thereby confounding mechanistic studies of pathogenesis. Consequently, virulence pathways utilized by HA-MRSA in nosocomial pneumonia are largely unknown. Here, we report that conditioning mice with broad-spectrum antibiotics lowers the barrier to pneumonia, thereby transforming otherwise avirulent HA-MRSA isolates into lethal pathogens. HA-MRSA isolates are avirulent in gnotobiotic mice, mimicking results in conventional animals. Thus, the observed enhanced susceptibility to infection in antibiotic-treated mice is not due to depletion of the microbiota. More generally, we found that antibiotic conditioning leads to increased susceptibility to infection by diverse antimicrobial-resistant (AMR) pathogens of low virulence. Treatment with antibiotics leads to dehydration and malnutrition, suggesting a potential role for these clinically relevant and reducible hospital complications in susceptibility to pathogens. In sum, the model described here mitigates the impact of low virulence in immunocompetent mice, providing a convenient model to gain fundamental insight into the pathogenesis of nosocomial pathogens.

## INTRODUCTION

Staphylococcus aureus is responsible for a large number of nosocomial and community-acquired infections worldwide. Methicillin-resistant S. aureus (MRSA) is among the most common causes of health care-associated pneumonia, accounting for up to 40% of hospital-acquired (1) and ventilator-associated (2) pneumonia subsets. Mortality rates of MRSA pneumonia are over 20%, even with optimal clinical intervention (2). Currently, there is no licensed vaccine or biologic to prevent or combat MRSA infections, which have become increasingly frequent since the mid-1990s. To confront the growing problem of MRSA, we require a greater understanding of pathobiology during infection, which remains poorly understood partly due to the lack of *in vivo* models relevant to the MRSA strains that infect hospitalized patients.

Most research to date has focused on highly virulent and cytotoxic community-acquired MRSA (CA-MRSA) strains; however, nosocomial pneumonia infections are most commonly caused by hospital-acquired MRSA (HA-MRSA) isolates, which exhibit low cytotoxicity toward human neutrophils in *ex vivo* models of infection (2, 3). Moreover, many of the nosocomial isolates responsible for morbidity and mortality in humans exhibit low virulence in commonly used preclinical animal models (4, 5). HA-MRSA is uncommon in the community, suggesting that these isolates cannot overcome the immune barriers of healthy individuals. The gene contents of HA-MRSA and CA-MRSA isolates differ (6), and many CA-MRSA virulence factors associated with acute infection in healthy hosts are considered dispensable for virulence in hospital environments (2, 7). Examination of the virulence strategies specifically used by HA-MRSA isolates, which is currently lacking, may be of the utmost relevance for infections of hospitalized patients.

The study of MRSA isolates associated with nosocomial infections has been complicated by the lack of susceptible *in vivo* models as these strains exhibit very low virulence in commonly used preclinical animal models of infection. We discovered that antibiotic treatment of mice increases susceptibility to HA-MRSA infection, thereby enabling pathogenesis studies that rely on demonstrating toxic or lethal effects in animals. Antibiotic treatment of mice is associated with dehydration and malnutrition, resulting in weight loss. This antibiotic treatment model allows us to work with clinical isolates that cause lethal infections in humans but display very weak virulence profiles in tissue culture models as well as regular *in vivo* murine models of infection. We expect that the use of this “hospital-adapted” nosocomial mouse model will aid in the identification of virulence factors involved in the pathogenesis of HA-MRSA and other low-virulence pathogens.

## RESULTS

### HA-MRSA pneumonia isolates exhibit low virulence *in vitro* and *in vivo*.

Recent work indicates that HA-MRSA isolates of low cytotoxicity are associated with mortality during nosocomial pneumonia in humans (2). We examined the cytotoxicity profiles of two representative HA-MRSA isolates, TH16 and BS_4884, which were obtained from patients with lethal hospital-acquired pneumonia (8). Whole-genome sequencing (WGS) and subsequent genomics analysis showed that TH16 is a clonal complex 8 (CC8) variant of the USA300 epidemic clone, with 99.95% average nucleotide identity (ANI) to the CA-MRSA strain FPR3757 (see Fig. S1B in the supplemental material). BS_4884, on the other hand, is part of the CC5 lineage (Fig. S1C) and is closely related to strain AR465 (99.96% ANI) (Fig. S1C). The CC8 and CC5 lineages encompass some of the most prevalent clones causing hospital-associated infections (6, 9, 10).

10.1128/mbio.01240-22.1 FIG S1 Comparative genomics of TH16 and BS_4884. (A) Phylogenetic placement of TH16 and BS_4884 (in red) among selected reference assemblies available in the NCBI RefSeq database. The tree was constructed using RAxML. (B and C) Comparison of TH16 to FPR3757 (B) and of BS_4884 to AR465 (C). From the center to the outside, the GC content is indicated in black. Prophages predicted using VirSorter2 are shown in dark green, and those predicted using Phigaro are shown in light green. Genes in the reference strain are shown in blue and red to indicate plus and minus strands, respectively. Contigs of the reference assembly are represented with gray ring segments. Genes present in the reference strain but absent in the query strain are shown in purple. The red ring shows 10-kbp windows from the reference assembly that mapped to the novel comparison with a BLAST E value of <10^−20^. (D) Results of a sequence similarity search for the protein sequences of 10 toxin genes. Black squares represent hits with >99% query coverage and >99% amino acid sequence identity. Download FIG S1, TIF file, 1.3 MB. Copyright © 2022 Lacey et al. 2022 Lacey et al. https://creativecommons.org/licenses/by/4.0/ This content is distributed under the terms of the Creative Commons Attribution 4.0 International license.

**FIG 1:**
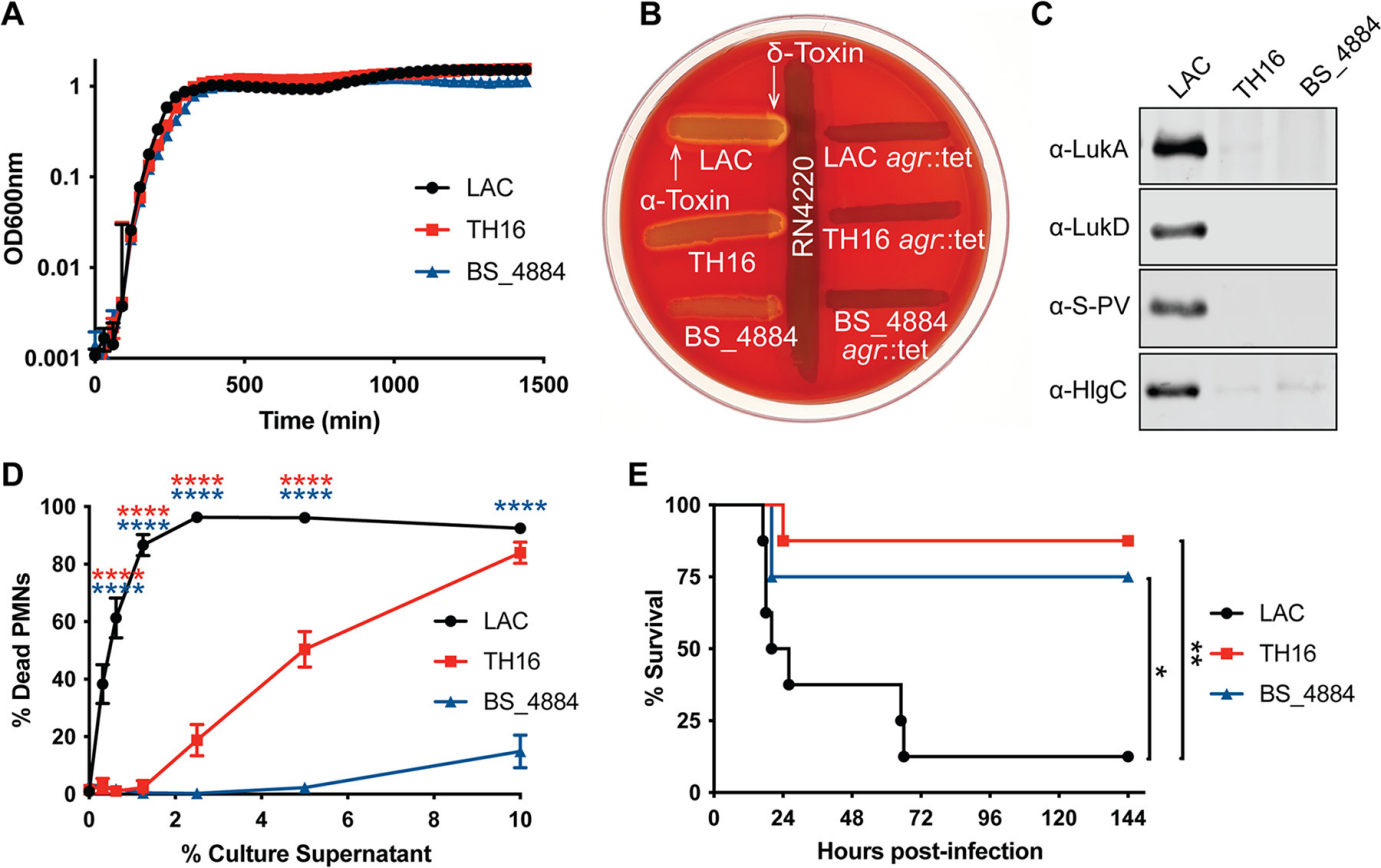
Virulence profile of HA-MRSA. (A) Growth curves of S. aureus strains LAC, TH16, and BS_4884 (*n* = 3) (means ± standard errors of the means [SEM]). (B) Strains were cross-streaked against RN4220 on sheep blood agar plates. (C) Immunoblot probing with antibodies against four different leukotoxins, after LAC, TH16, and BS_4884 had been grown in TSB for 5 h. (D) Human neutrophils were exposed to various concentrations of culture supernatants isolated after 5 h, and cell death was measured using the CellTiter assay (*n* = 8 donors) (means ± SEM). (E) Survival curve of C57BL/6J mice challenged intranasally with 4 × 10^8^ CFU (*n* = 8 mice/group). Statistical analysis was performed using two-way analysis of variance (ANOVA) with Dunnett’s posttest (D) or a log rank (Mantel-Cox) test (E). *, *P* < 0.05; **, *P* < 0.01; ****, *P* < 0.0001.

For control and comparison, we used the well-characterized and highly cytotoxic CA-MRSA USA300 strain LAC (11). There was no difference in growth in rich medium among LAC, TH16, and BS_4884 (Fig. 1A). The production of alpha- and delta-toxins was investigated by cross-streaking onto sheep blood agar against the S. aureus laboratory strain RN4220. While delta-hemolysin is weakly active on sheep blood agar on its own, it synergizes strongly with beta-hemolysin, the only hemolysin produced by RN4220 (12), resulting in a zone of clear hemolysis. The hemolytic patterns of the strains were indicative of lower levels of alpha- and delta-toxin production by TH16 and BS_4884 than by LAC (Fig. 1B). S. aureus can produce a number of bicomponent, pore-forming leukocidins, which target and kill a wide variety of primary human leukocytes critical for innate and adaptive immune functions, including neutrophils, monocytes, macrophages, dendritic cells, and effector memory T cells (13–20). The presence of LukAB, LukED, LukSF-PV, (Panton-Valentine leucocidin [PVL]), and HlgAB in the supernatant after 5 h of growth *in vitro* by isolates TH16 and BS_4884 was negligible compared to that of LAC as determined by immunoblotting (Fig. 1C). In accordance with these results, when primary human neutrophils were exposed to culture supernatants from these strains, BS_4884 was incapable of causing cell death, and TH16 was unable to cause cell death at low concentrations, in contrast to LAC, which was highly cytotoxic at all concentrations (Fig. 1D). Of note, WGS analysis of these isolates showed that these strains harbor the toxin loci and that the predicted protein sequences of Hla, HlgA, HlgB, HlgC, LukA, LukB, LukD, and LukE in these isolates were >99% identical to those of LAC (Fig. S1D). LukF-PV and LukS-PV, while predicted to be absent in BS_4884, showed >99% sequence identity in TH16 compared to LAC.

Next, we used a pneumonia model of infection to investigate the virulence potential of these strains *in vivo*. As expected, mice challenged with LAC succumbed to infection rapidly (21), while mice challenged with the HA-MRSA isolates survived for the most part (Fig. 1E). Taken together, these results indicate that HA-MRSA isolates, which are often lethal in respiratory tract infections in humans (2), exhibit low cytotoxicity *in vitro* and low virulence potential in conventional *in vivo* models of infection.

### Antibiotic treatment sensitizes mice to HA-MRSA infection.

The intestinal microbiota is of great clinical relevance, particularly in hospital settings where the majority of patients are treated with antibiotics, which disrupt the microbiota and promote dysbiosis (22). It was previously demonstrated that antibiotic preexposure leads to accelerated mortality and increased bacterial burdens in the lungs of mice challenged with Streptococcus pneumoniae (23), Burkholderia pseudomallei (24), and Klebsiella pneumoniae (25). Nevertheless, there is a paucity of information regarding the protective role of the intestinal microbiota against MRSA infection at distal sites (26) and the mechanism of increased susceptibility to these pathogens. Accordingly, we sought to explore the effects of antibiotic exposure on susceptibility to the pathogenesis of low-virulence HA-MRSA in pneumonia.

**FIG 2:**
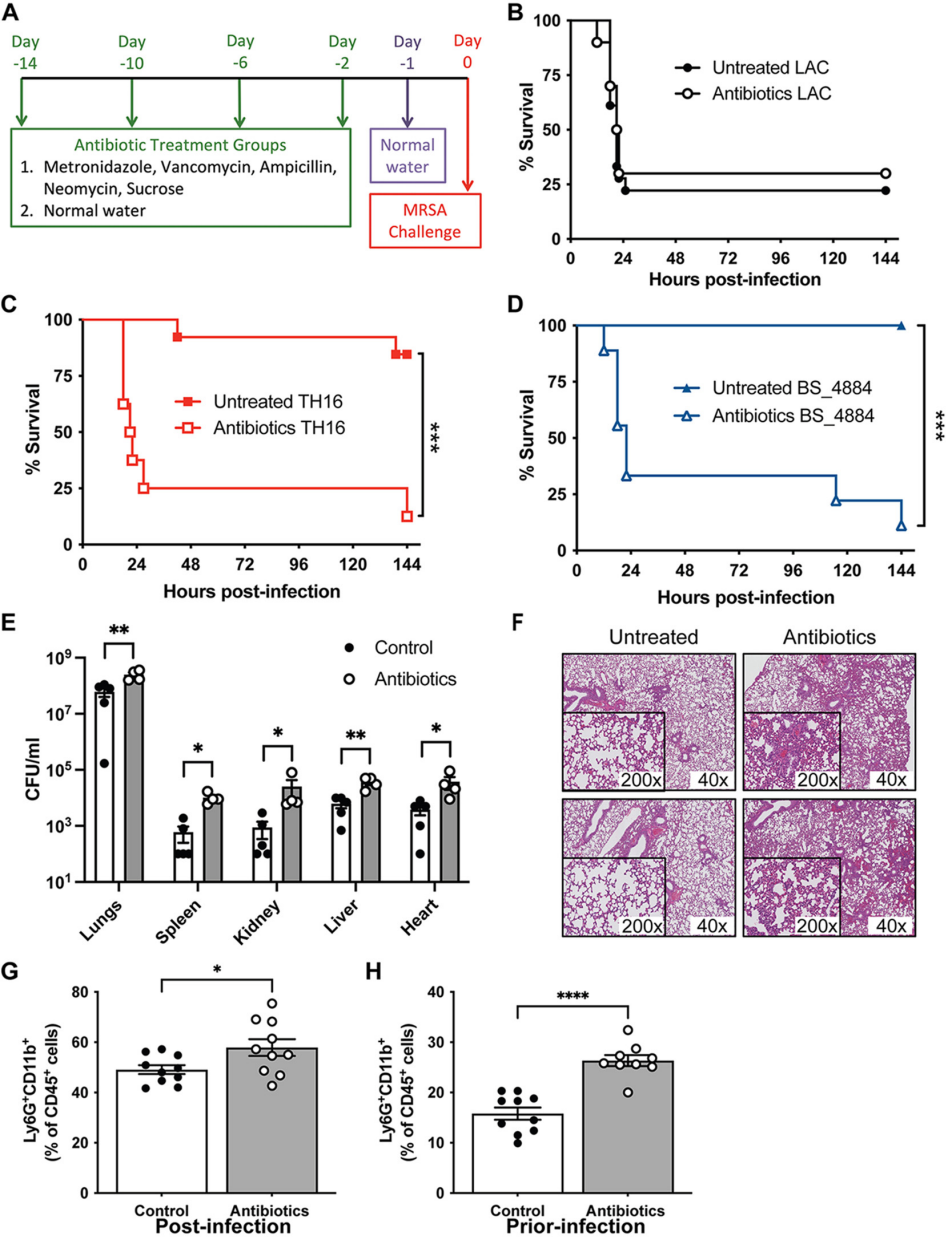
Antibiotic treatment sensitizes mice to HA-MRSA infection. (A) C57BL/6J mice were preexposed to metronidazole (1 g/L), vancomycin (0.5 g/L), ampicillin (1 g/L), and neomycin (0.5 g/L) in their drinking water for 2 weeks and switched to antibiotic-free water 1 day prior to challenge. (B to D) Survival curves of intranasal infection with 4 × 10^8^ CFU of LAC (B), TH16 (C), and BS_4884 (D) (*n* = 8 to 18 mice/group). (E) Bacterial burden at 4 h postinfection in the lung and peripheral organs of mice infected with TH16 (*n* = 4 to 5 mice/group). (F) H&E staining of two representative histological sections of lung tissue 18 h after infection with 1 × 10^8^ CFU of TH16. (G and H) Flow cytometry analysis of lung tissue 18 h after infection with1 × 10^8^ CFU of TH16 (G) and prior to infection (H). Neutrophils were gated as live, single, TCRβ^−^ B220^−^ CD45^+^ Ly6G^+^ CD11b^+^ cells. Statistical analysis was performed using a log rank (Mantel-Cox) test (B to D), two-way ANOVA with a Šídák posttest (E), and Student’s *t* test (G and H). *, *P* < 0.05; **, *P* < 0.01; ***, *P* < 0.001.

Mice were given a cocktail of antibiotics in their drinking water for 13 days and switched to antibiotic-free water 1 day prior to infection (Fig. 2A). While challenging untreated mice intranasally (i.n.) with CA-MRSA strain LAC resulted in over 75% mortality (Fig. 2B), almost all untreated mice challenged with HA-MRSA strains TH16 (Fig. 2C) and BS_4884 (Fig. 2D) survived. Remarkably, ~90% of mice preexposed to antibiotics and infected with HA-MRSA strains succumbed to infection (Fig. 2C and D). In contrast, antibiotic exposure had no effect on mice infected with LAC (Fig. 2B).

We next sought to investigate the susceptibility of antibiotic-treated mice further and used TH16 as a representative HA-MRSA isolate. The bacterial burden in the lungs of antibiotic-treated mice challenged with TH16 was significantly increased at 4 h postinfection (hpi) compared to untreated mice (Fig. 2E). The bacterial loads in the spleen, liver, kidneys, and heart were also elevated in antibiotic-treated animals, indicating that antibiotic treatment increases dissemination to the peripheral organs. Tissue sections from lungs of mice infected intranasally with a nonlethal dose of TH16 showed considerable differences in the host response to HA-MRSA between untreated and antibiotic-treated mice (Fig. 2F). The lungs of antibiotic-treated mice were visibly more inflamed, with the alveoli containing large numbers of granulocytic cells. Using flow cytometry, lung tissue from antibiotic-treated mice infected with TH16 was found to have significantly increased numbers of neutrophils compared to control mice 18 h after nonlethal infection (Fig. 2G). Interestingly, antibiotic treatment alone (day 0, prior to infection) led to an increase in the number of neutrophils present in the lung tissue (Fig. 1H). This suggests that increased numbers of neutrophils in the antibiotic-treated host may result in a negative effect on infection outcomes with HA-MRSA, likely due to an overzealous neutrophil response, which has previously been associated with epithelial damage (27–29). Previous studies have demonstrated that staphylococcal protein A is highly proinflammatory in the context of pneumonia (27, 30). However, there was no difference in the levels of protein A produced *in vitro* by LAC, TH16, and BS_4884, as measured by Western immunoblotting (Fig. S2).

10.1128/mbio.01240-22.2 FIG S2 Protein A levels are the same between strains. Immunoblot probing was performed for protein A of LAC, TH16, and BS_4884 grown in TSB for 5 h. The band intensity was quantified using ImageJ, and the results are presented as integrated density (Int/Den). A representative immunoblot is presented below. Download FIG S2, TIF file, 1.5 MB. Copyright © 2022 Lacey et al. 2022 Lacey et al. https://creativecommons.org/licenses/by/4.0/ This content is distributed under the terms of the Creative Commons Attribution 4.0 International license.

In sum, exposure to antibiotics can mitigate the impact of reduced bacterial virulence in mice, allowing a permissive environment for HA-MRSA isolates. However, this does not affect highly cytotoxic CA-MRSA strains, which are equally virulent in treated and untreated mice. These findings establish a model that enables us to probe the bacterial and host factors important during nosocomial infections.

### Genes regulated by *agr* are responsible for severe morbidity during nosocomial pneumonia.

**FIG 3:**
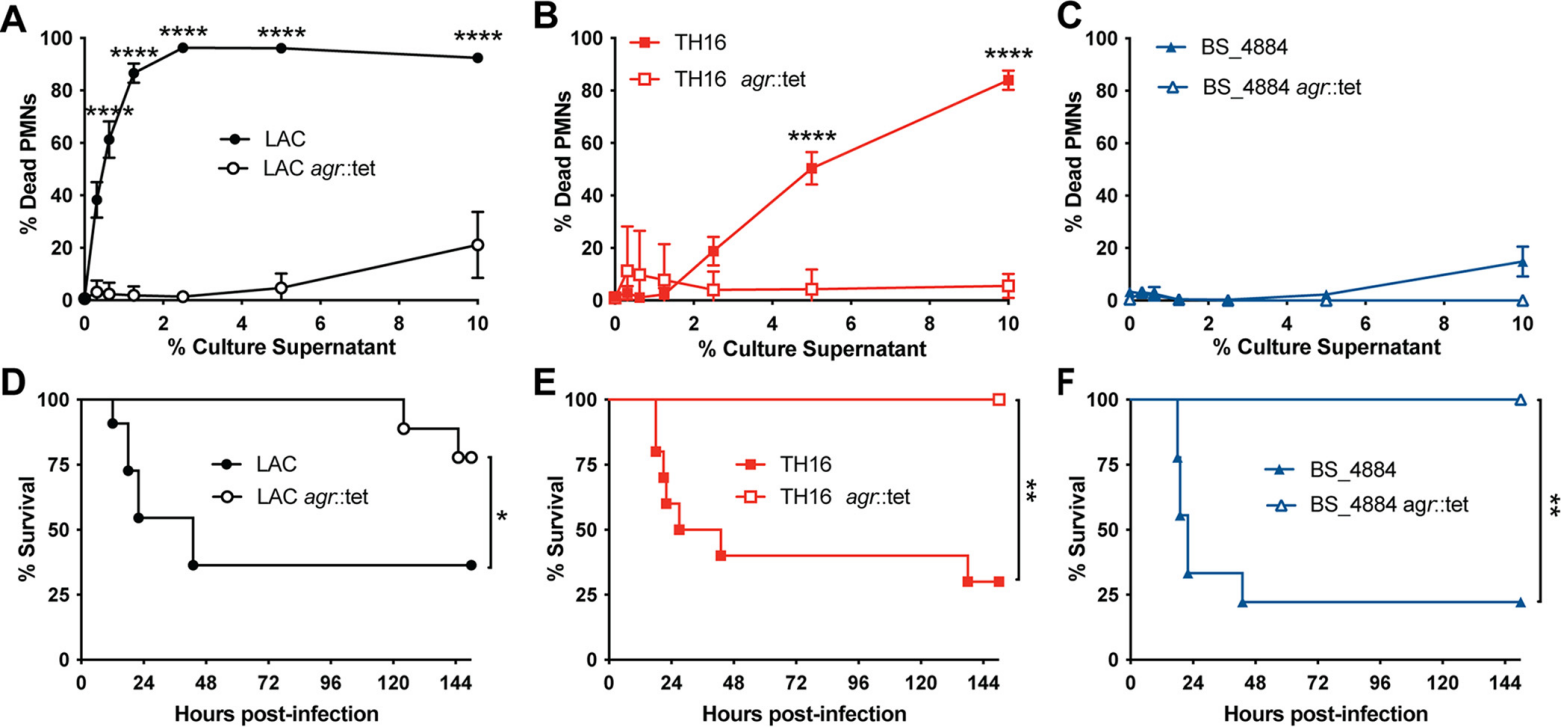
Virulence factors regulated by *agr* are responsible for severe morbidity during nosocomial pneumonia. (A to C) Human neutrophils were exposed to 5-h culture supernatants from LAC (A), TH16 (B), and BS_4884 (C), and cell death was measured using the CellTiter assay (*n* = 7 to 8 donors/group) (means ± SEM). (D to F) Survival curves of antibiotic-treated C57BL/J mice infected i.n. with LAC (D), TH16 (E), and BS_4884 (F) or isogenic *agr* mutants of these strains (*n* = 6 to 11 mice/group). Statistical analysis was performed using two-way ANOVA with a Šídák posttest (A to C) and a log rank (Mantel-Cox) test (D to F). *, *P* < 0.05; **, *P* < 0.01; ****, *P* < 0.0001.

It is unclear how HA-MRSA strains cause infection in humans. Chief among the regulators of virulence in S. aureus is the Agr two-component quorum sensing system (31, 32). To determine whether genes regulated by Agr were required for the virulence of HA-MRSA isolates, isogenic mutants of *agr* in LAC, TH16, and BS_884 were constructed and verified (Fig. 1B). Culture supernatants from *agr* mutants of LAC (Fig. 3A), TH16 (Fig. 3B), and BS_4884 (Fig. 3C) were incapable of killing human neutrophils, in contrast to their parental strains. We next infected antibiotic-treated mice with these strains and monitored morbidity as a measure of acute infection. When antibiotic-treated mice were challenged intranasally with *agr* mutants, the mice survived infection regardless of the lineage. Thus, although HA-MRSA isolates often exhibit low levels of Agr activity *in vitro* (5, 33–37), the observed antibiotic-dependent virulence in mice infected with TH16 (Fig. 3E) and BS_4884 (Fig. 3F) is *agr* dependent. These data establish that Agr-regulated genes are responsible for causing severe morbidity and mortality by HA-MRSA in antibiotic-treated mice.

### Antibiotic treatment sensitizes mice to bloodstream infections with all ESKAPE pathogens.

**FIG 4:**
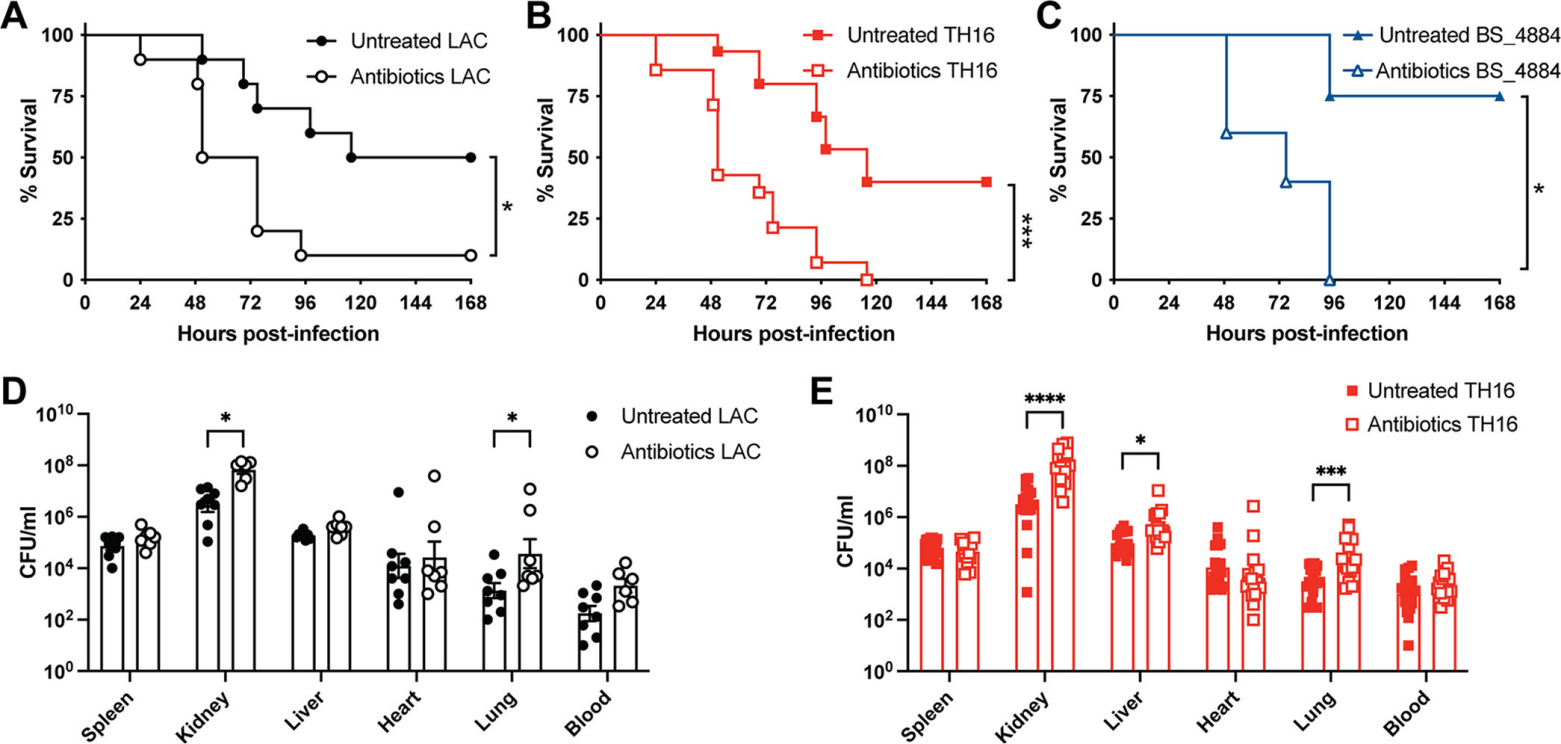
Antibiotic treatment sensitizes mice to S. aureus bloodstream infection. (A to C) Survival curves of antibiotic-treated C57BL/6J mice infected intravenously (i.v.) with 1 × 10^7^ CFU of LAC (A), TH16 (B), and BS_4884 (C) (*n* = 5 to 15 mice/group). (D and E) Bacterial burdens of intravenously infected mice 24 h after infection with LAC (D) and TH16 (E) (*n* = 7 to 18 mice/group) (means ± SEM). Statistical analysis was performed using a log rank (Mantel-Cox) test (A to C) and two-way ANOVA with a Šídák posttest (D and E). *, *P* < 0.05; ***, *P* < 0.001.

Having demonstrated that antibiotic treatment can sensitize mice to pneumonia caused by HA-MRSA clinical isolates, we next investigated whether this model could also be used to study hospital-acquired infections other than pneumonia. As S. aureus is one of the leading causes of bloodstream infection (BSI) and carries a higher mortality rate than any other causes of bacteremia despite appropriate treatments (38), we treated mice with antibiotics prior to BSI by low- and high-virulence MRSA strains. Antibiotic-treated mice showed greater mortality after BSI challenge with LAC (Fig. 4A), TH16 (Fig. 4B), and BS_4884 (Fig. 4C) than untreated mice. This significant increase in mortality was accompanied by an increase in the bacterial burden at 24 hpi in the organs of mice infected with LAC (Fig. 4D) and TH16 (Fig. 4E).

**FIG 5:**
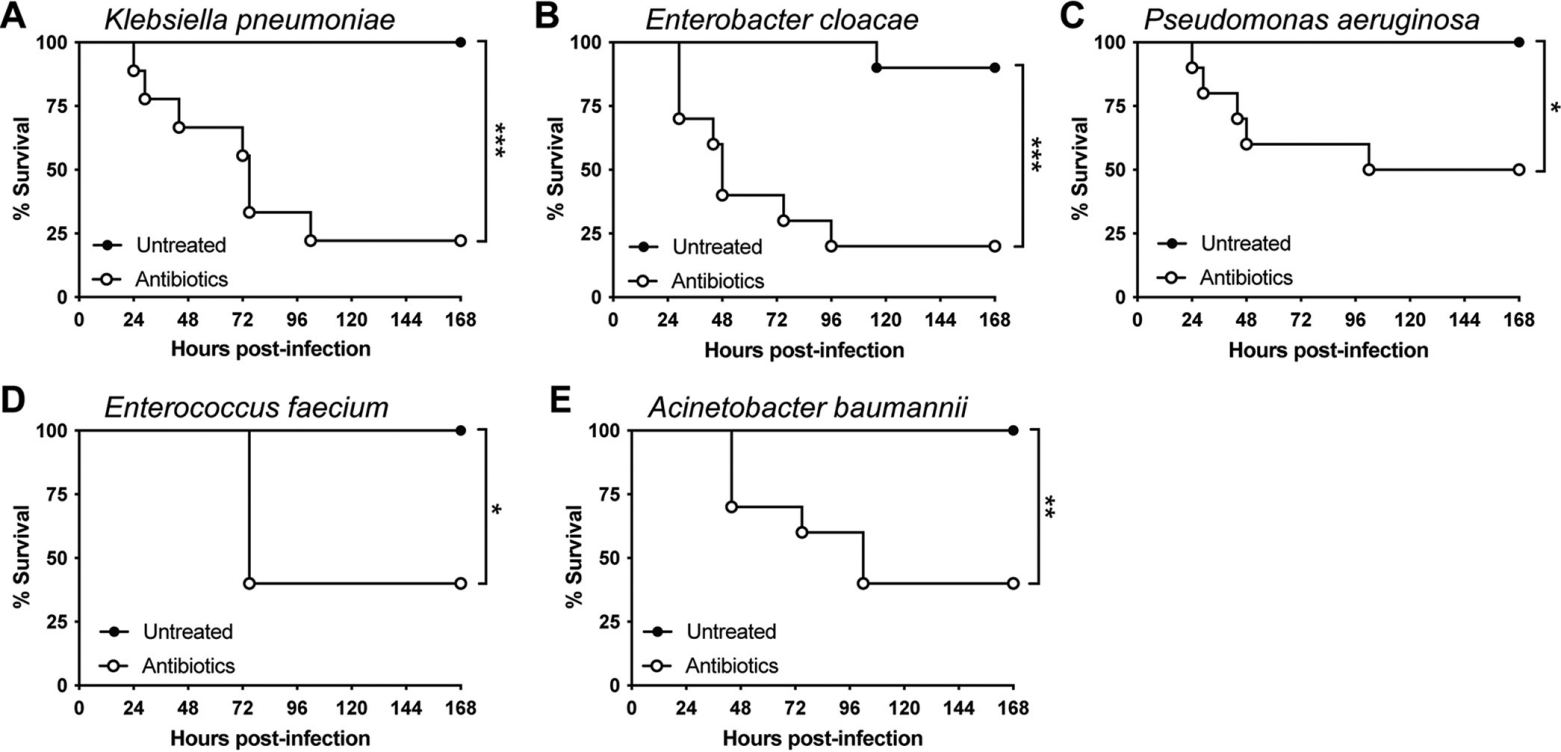
Antibiotic treatment sensitizes mice to bloodstream infection with ESKAPE pathogens. Shown are survival curves of antibiotic-treated C57BL/6 mice infected i.v. with 5 × 10^7^ CFU of clinical isolates of Klebsiella pneumoniae (A), the Enterobacter cloacae complex (B), Pseudomonas aeruginosa (C), Enterococcus faecium (D), and Acinetobacter baumannii (E) (*n* = 5 to 10 mice/group). Statistical analysis was performed using a log-rank (Mantel-Cox) test. *, *P* < 0.05; ***, *P* < 0.001.

Given that our antibiotic treatment model sensitized mice to BSI with MRSA, we went on to evaluate the virulence of other members of the ESKAPE group of antimicrobial-resistant (AMR) pathogens (Enterococcus faecium, Staphylococcus aureus, Klebsiella pneumonia, Acinetobacter baumannii, Pseudomonas aeruginosa, and Enterobacter species) (39). We found that antibiotic-treated mice rapidly succumb to BSI with representative clinical isolates of all ESKAPE group members (Fig. 5A to E). Remarkably, none of these pathogens were lethal in untreated control mice. Taken together, these results highlight the broad application of this model to study AMR pathogens using multiple models of infection.

### Increased susceptibility to S. aureus is microbiome independent.

**FIG 6:**
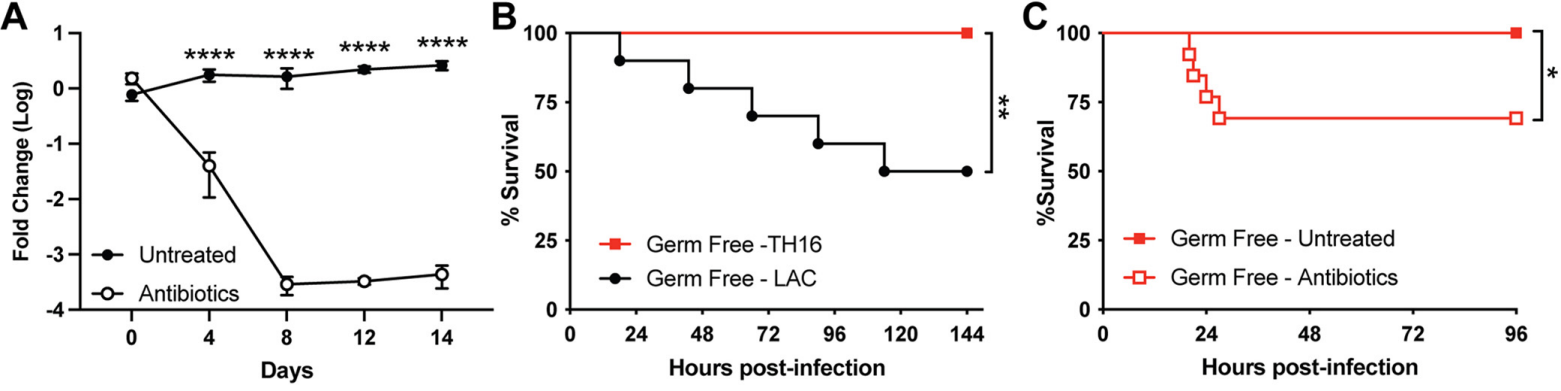
Increased susceptibility to S. aureus in antibiotic-treated mice is microbiome independent. (A) The total microbial load of stool was measured by 16S sequencing of DNA extracted from stool samples of untreated and antibiotic-treated mice (*n* = 5) (means ± SEM). (B) Survival curve of germfree mice challenged intranasally with 4 × 10^8^ CFU of LAC or TH16 (*n* = 10 to 11 mice/group). (C) Survival curve of antibiotic-treated germfree mice infected intranasally with 4 × 10^8^ CFU of TH16 (*n* = 12 to 13 mice/group). Statistical analysis was performed using two-way ANOVA with a Šídák posttest (A) and a log rank (Mantel-Cox) test (B and C). *, *P* < 0.05; **, *P* < 0.01; ****, *P* < 0.0001.

The intestinal microbiota is crucial for the host defense system, supporting mucosal immunity and modulating systemic immunity (22–24). As our model utilizes antibiotics, we investigated if changes in the host microbiome were the cause of the increased susceptibility to low-virulence nosocomial pathogens. Stool samples were collected throughout the 14-day antibiotic treatment period, DNA was isolated, and quantitative PCR (qPCR) was performed to determine the microbial load in the intestines of these mice. As expected, the microbial load was significantly diminished during antibiotic treatment (Fig. 6A). To determine if this disruption of the microbiome was responsible for the increased susceptibility to MRSA, we challenged germfree mice, which lack all microorganisms, intranasally with LAC and TH16 (Fig. 6B). Germfree mice succumbed to pneumonia challenge with LAC but were resistant to TH16, mirroring the results in conventional mice. Subjecting germfree mice to the oral antibiotic regimen (Fig. 2A) sensitized the mice to TH16 infection (Fig. 6C). These results indicate that disruption of the microbiome is insufficient to explain the increased susceptibility to MRSA caused by antibiotics.

### Oral antibiotics lead to temporary weight loss due to reduced water and calorie intake.

**FIG 7:**
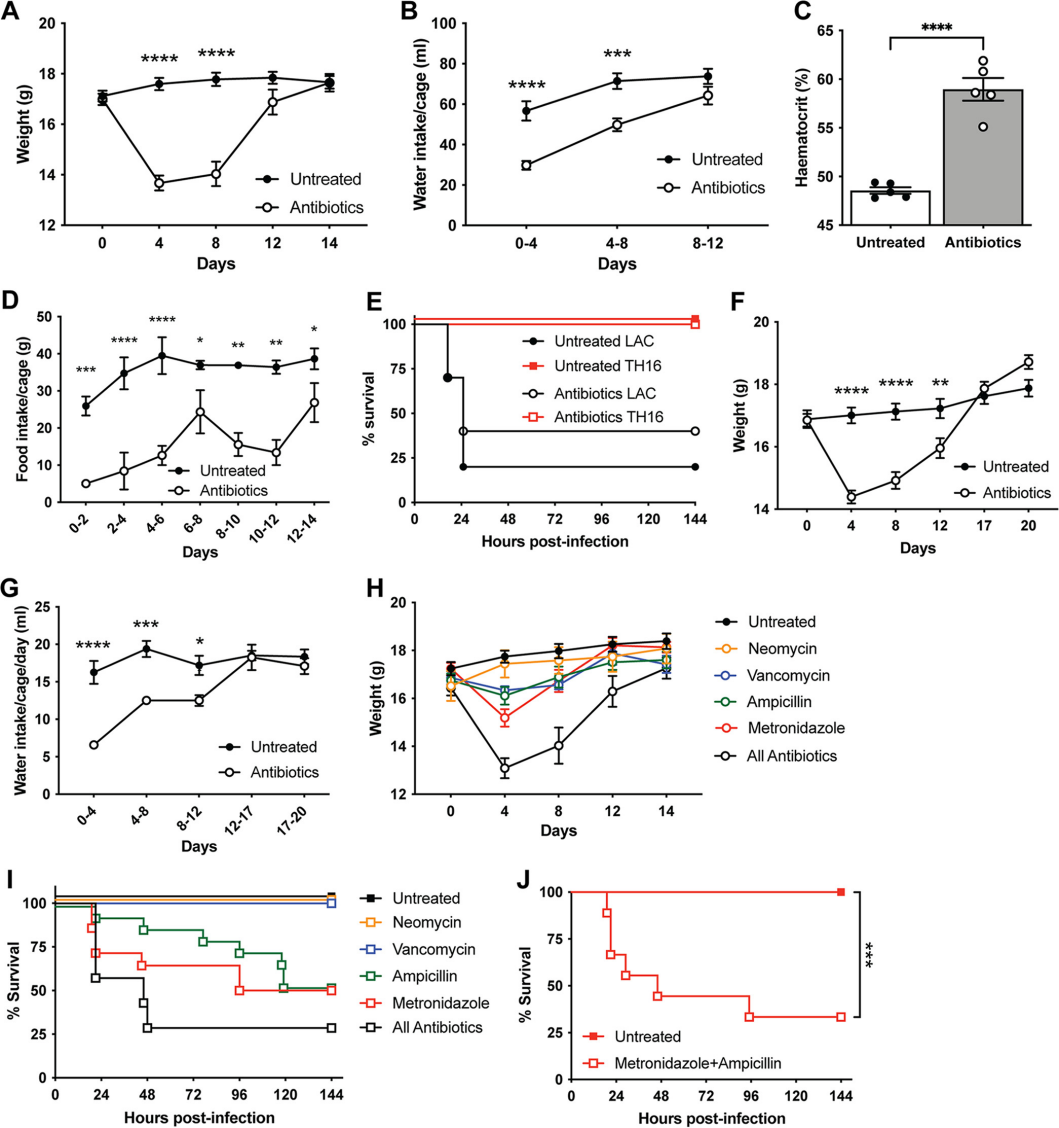
Antibiotics in drinking water lead to temporary weight loss due to reduced water and calorie intake. (A, B, and D) C57BL/6J mice were treated with antibiotics for 2 weeks, and weight (*n* = 24 mice/group) (means ± SEM) (A), water intake (*n* = 7 to 20 cages/group) (means ± SEM) (B), and food intake (*n* = 6 cages/group) (means ± SEM) (D) were recorded. (C) Mice were bled at day 4, and hematocrit was measured (*n* = 5 mice/group) (means ± SEM). (E) Survival curve of mice treated with antibiotics for 3 weeks and challenged intranasally with 4 × 10^8^ CFU of TH16 (*n* = 5 to 10 mice/group). (F and G) Weight (*n* = 20 mice/group) (means ± SEM) (F) and water intake (*n* = 4 cages/group) (means ± SEM) (G) of mice treated with antibiotics for 3 weeks. (H) Weight of mice treated with individual antibiotics (*n* = 5 to 15 mice/group) (means ± SEM). (I) Survival curve of mice treated with individual antibiotics and challenged intranasally with 4 × 10^8^ CFU of TH16 (*n* = 5 to 15 mice/group). (J) Survival curve of mice treated with metronidazole (1 g/L) and ampicillin (1 g/L) in their drinking water for 2 weeks and switched to antibiotic-free water 1 day prior to challenge. Mice were challenged intranasally with 4 × 10^8^ CFU of TH16 (*n* = 5 to 15 mice/group). Statistical analysis was performed using two-way ANOVA with a Šídák posttest (A, B, D, F, G, and J), Student’s *t* test (C), and a log rank (Mantel-Cox) test (J). *, *P* < 0.05; **, *P* < 0.005; ***, *P* < 0.001; ****, *P* < 0.0001.

Having determined that our phenotype was occurring independently of the microbiome, we further investigated the effects of antibiotic treatment on mice. We observed that mice lost significant weight initially during antibiotic exposure over the 2-week period prior to infection (Fig. 7A). Weight loss was also seen in germfree mice treated with antibiotics (Fig. S3). To investigate the cause of weight loss, we measured water and food consumption. Water intake during the antibiotic treatment period was found to be significantly reduced in the antibiotic-treated groups compared to the untreated groups (Fig. 7B). Antibiotic-treated mice also exhibited elevated hematocrit (Fig. 7C), consistent with dehydration, on day 4 of antibiotic treatment. Antibiotic-treated mice also had reduced food intake compared to control animals (Fig. 7D). Importantly, mice adapt to antibiotics in their diet and begin to refeed and gain weight, returning to the same weight as that of control mice by day 14. Consistent with this recovery, when we treated mice with antibiotics for 3 weeks, they were no longer susceptible to TH16 pneumonia infection but still succumbed to infection with LAC (Fig. 7E). Mice treated with antibiotics regained weight (Fig. 7F), and their water intake (Fig. 7G) also returned to control levels by day 14 of treatment. These data suggest that the effect of weight loss on infection is temporary and that upon refeeding, mice can revert to baseline resistance to infection after a period of time.

10.1128/mbio.01240-22.3 FIG S3 Germfree mice also lose weight when exposed to antibiotics in their drinking water. Weights of germfree mice treated with antibiotics were determined (*n* = 12 to 13 mice/group) (means ± SEM). Statistical analysis was performed using two-way ANOVA with a Šídák posttest. ****, *P* < 0.0001. Download FIG S3, TIF file, 1.7 MB. Copyright © 2022 Lacey et al. 2022 Lacey et al. https://creativecommons.org/licenses/by/4.0/ This content is distributed under the terms of the Creative Commons Attribution 4.0 International license.

To determine if any one antibiotic was responsible for weight loss, we treated mice with each antibiotic individually. Mice lost differing amounts of weight depending on the antibiotic with which they were treated (Fig. 7H). For example, mice treated with metronidazole and ampicillin lost more weight than mice treated with vancomycin and neomycin (Fig. 7H). When mice were challenged intranasally with TH16, they succumbed to infection when treated with metronidazole or ampicillin but not vancomycin or neomycin (Fig. 7I). Additionally, mice treated with a combination of metronidazole and ampicillin succumbed to TH16-mediated pneumonia (Fig. 7J), phenocopying treatment with all four antibiotics. Thus, we observe an inverse correlation between weight loss and survival, where greater weight loss leads to increased susceptibility to HA-MRSA pneumonia.

### Antibiotic treatment does not cause ablation of phagocytes.

**FIG 8:**
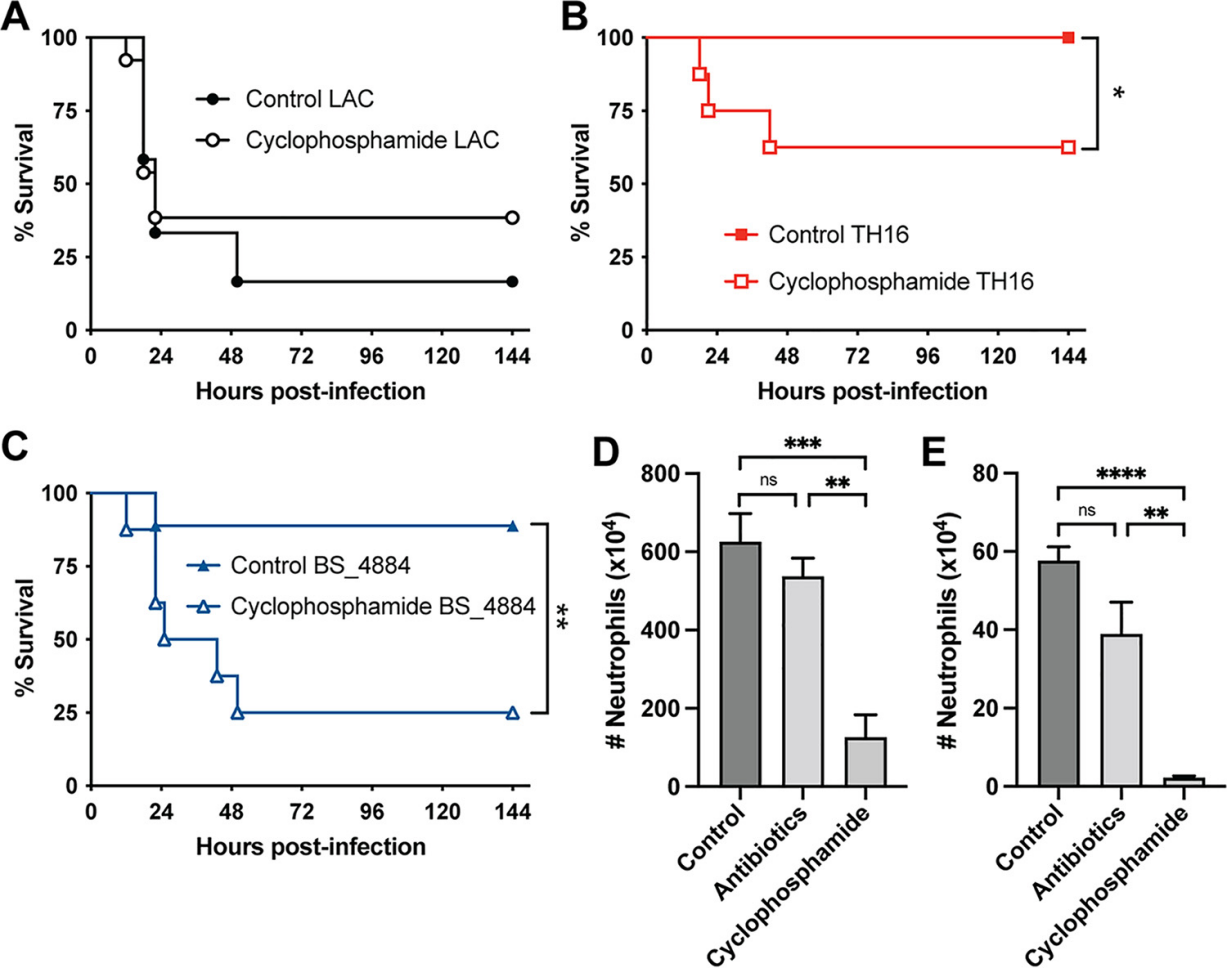
Antibiotics in drinking water increased susceptibility to infection without ablating neutrophils. (A to C) C57BL/6J mice were treated with cyclophosphamide intraperitoneally for 1 week and subsequently infected intranasally with LAC (A), TH16 (B), and BS_4884 (C), and survival was measured (*n* = 8 to 13 mice/group). (D and E) The numbers of neutrophils in the bone marrow (D) and the spleen (E) were quantified using flow cytometry (*n* = 4 to 5 mice/group) (means ± SEM). Statistical analysis was performed using a log rank (Mantel-Cox) test (A to C) and two-way ANOVA with a Šídák posttest (D and E). *, *P* < 0.05; **, *P* < 0.005; ***, *P* < 0.001; ****, *P* < 0.0001; ns, not significant.

Neutrophil depletion can increase the sensitivity of mice to S. aureus infection (40–42). Indeed, humans with neutrophil deficiencies suffer from increased S. aureus infections (43–45). Treating mice with cyclophosphamide, a bone marrow-ablating chemotherapy agent, induces neutropenia and sensitizes mice to S. aureus infection (46), as seen in human patients (47, 48). Cyclophosphamide treatment mirrored the results seen with the antibiotic treatment model where it sensitized the mice to infections with TH16 (Fig. 8B) and BS_4884 (Fig. 8C) but not LAC (Fig. 8A). However, cyclophosphamide-treated mice are severely immunocompromised and have significantly reduced numbers of neutrophils in the bone marrow (Fig. 8D) and spleen (Fig. 8E). In contrast, antibiotic-treated mice retain levels of neutrophils in the bone marrow and spleen similar to those in control mice. Thus, the model described here provides an immunocompetent *in vivo* model to study ESKAPE pathogens that otherwise exhibit low virulence in conventional murine models.

## DISCUSSION

The study of HA-MRSA pathogenesis has been confounded by a lack of *in vivo* models that are susceptible to infection by these “low-virulence” organisms. Previous studies from our group showed that HA-MRSA isolates are associated with increased mortality in patients with nosocomial pneumonia (2). Here, we demonstrate that representative isolates from the two major HA-MRSA lineages in the United States (CC8 and CC5) exhibit low-cytotoxicity phenotypes in *in vitro* and *ex vivo* models of intoxication and infection of human neutrophils, consistent with the observation that HA-MRSA isolates often produce low levels of toxins (3, 5, 49). Using an established pneumonia model of infection (21, 50, 51), HA-MRSA isolates were avirulent, in contrast to CA-MRSA. Together, our data highlight important differences between CA-MRSA and HA-MRSA interactions with the host.

AMR pathogens currently pose one of the greatest threats to human health, with the global death toll from drug-resistant infections estimated to be at least 700,000 annually (52). While efforts to develop antibacterial drugs and vaccines are crucial to mitigate the threat of AMR, new models to study these infections provide the building blocks for discovering novel drug targets and vaccine antigens. Our antibiotic treatment model was also able to increase susceptibility to all of the AMR pathogens in the ESKAPE group, demonstrating the wide application of this model for host-pathogen studies.

We identified that genes regulated by the global virulence regulator Agr are important determinants of morbidity and mortality in the antibiotic treatment model of pneumonia. Several Agr-regulated genes have been implicated in pneumonia, including the staphylococcal β-barrel pore-forming toxins (21, 50, 53–55). Interestingly, culture supernatants from strain BS_4884 were barely able to kill human polymorphonuclear leukocytes (PMNs) at high concentrations (Fig. 3C), yet the strain was nonetheless very virulent in both pneumonia and BSI models in nosocomial mice. These observations highlight the dichotomy between the *in vitro* and *in vivo* characteristics of HA-MRSA strains and underpin the importance of clinically relevant models when evaluating the virulence of MRSA strains.

A growing body of evidence suggests that the host microbiota influences local and systemic immunity. For example, germfree mice lack developed immune systems and have mucosal alterations that can be restored by colonization with gut microbiota (26, 56). This led us to initially hypothesize that the use of antibiotics may lead to changes in the ability of the host to respond to subsequent MRSA infection. Using a previously published protocol involving a cocktail of antibiotics in drinking water to deplete mice of their intestinal microbiota (57–59), we were able to reduce intestinal microbiota (Fig. 6C). However, we found that this treatment also led to increased susceptibility to HA-MRSA in a microbiome-independent manner. Consistent with this observation, germfree mice were no more susceptible to HA-MRSA than conventional mice, and germfree mice could be sensitized to infection by antibiotic treatment.

Collectively, our data suggest that antibiotics in the drinking water lead to dehydration and malnutrition in mice. We hypothesize that these perturbations stress the immune system, resulting in greater susceptibility to ESKAPE pathogens. Our data support previous studies that have found that oral antibiotic treatment leads to dehydration (58, 59). Mice refrain from drinking water containing antibiotics presumably due to the bitter taste of the antibiotics, particularly metronidazole (58), despite the addition of glucose as a sweetener. Thus, metronidazole, alone or together with ampicillin, is responsible for the effect. Notably, mice adapt to antibiotics and drink water to the same level as control mice after ~2 weeks, restoring resistance to infection. These data indicate that the above-described susceptibility to infection is temporal and can be reversed. The underlying mechanism(s) behind how dehydration and caloric reduction due to oral antibiotic treatment lead to hypersusceptibility to ESKAPE pathogens is being further characterized and is the focus of a future study.

Overall, the model described here provides an immunocompetent mouse model amenable for the study of the pathogenesis of ESKAPE pathogens in a host that more closely mimics hospitalized patients. We hope that this model will enable future studies aimed at the identification of novel targets for the development of preventives and therapeutics to combat nosocomial infections by ESKAPE pathogens.

## MATERIALS AND METHODS

### Bacterial strains and growth conditions.

S. aureus strains were grown on tryptic soy agar (TSA) or in tryptic soy broth (TSB). S. aureus cultures were grown in 5 mL of medium in 15-mL tubes with shaking at a 45° angle at 37°C. For all experiments, S. aureus was grown in TSB overnight, and a 1:100 dilution of the cultures grown overnight was subcultured into fresh TSB. Unless otherwise specified, S. aureus grown to early stationary phase (3 h) was collected and normalized by the optical density at 600 nm (OD_600_) for further experimental analysis.

Other ESKAPE microbes were grown overnight in brain heart infusion (BHI) broth, subcultured into fresh BHI broth at a 1:100 dilution, and grown for 3 h. The bacterial strains used in this study are listed in Table 1.

**TABLE 1:** Strains used in this study

Strain no.	Strain description	Reference(s)
VJT 12.61	S. aureus USA300 strain LAC; highly cytotoxic CA-MRSA CC8 clinical isolate	11
VJT 43.92	S. aureus USA300 strain TH16; low-cytotoxicity and epidemiologically HA-MRSA CC8 clinical isolate	2, 8
VJT 64.01	S. aureus strain BS_4884; low-cytotoxicity and epidemiologically HA-MRSA CC5 clinical isolate	2, 8
VJT 19.91	S. aureus LAC *agr*::*tet*	81
KAL 1.10	S. aureus TH16 *agr*::*tet*	This study
KAL 1.08	S. aureus BS_4884 *agr*::*tet*	This study
VJT 2.60	S. aureus strain RN4220	12
VJT 72.23	Klebsiella pneumoniae 161; clinical isolate	This study
VJT 72.18	Enterobacter cloacae 173; clinical isolate	This study
VJT 72.21	Pseudomonas aeruginosa 192; clinical isolate	This study
VJT 72.22	Enterococcus faecium T279; clinical isolate	This study
VJT 72.23	Acinetobacter baumannii 161; clinical isolate	This study

Phage 80α was used to transduce *agr*::*tet* into the indicated strains as previously described (60). Transductants were selected on TSA plates containing tetracycline.

### Genome sequencing, assembly, and annotation.

We prepared sequencing libraries from DNA extracted from each HA-MRSA isolate as previously described (61). Whole-genome sequencing was performed using an Illumina NovaSeq instrument, yielding paired 150-bp reads. Adapter sequences and low-quality bases were trimmed using fastp (62) v.0.20.1, using default parameters. Trimmed reads were checked for within-species contamination using ConFindr (63) v.0.7.4 and for cross-species contamination using MetaPhlAn (64) v.3.0.13. For the purposes of phylogenetic analysis, snippy (65) v.4.6.0 was used to generate a core-genome alignment of the trimmed reads and of selected RefSeq assemblies from CC5 and CC8, using an assembly of S. aureus strain FPR3757 (accession number GCF_000013465.1) as a reference.

Trimmed reads were assembled into contigs using Unicycler (66) v.0.4.8 with default parameters, in normal mode. Prokka (67) v.1.14.6 was used to identify and annotate genes in the resulting assemblies. The TH16 assembly was then compared to a reference assembly of strain FPR3757 (RefSeq accession number GCF_000013465.1), while the BS_4884 assembly was compared to a reference assembly of strain AR465 (accession number GCF_003073655.1). For this purpose, reference assemblies were divided into 10-kbp windows. Each window was used in a BLASTn (version 2.12.0) query (68) against the newly sequenced assembly (FPR3757 versus TH16 and AR465 versus BS_4884), keeping only alignments with an E value of <10^−20^. The GC content [(G+C)/(A+T+G+C)] for each window was calculated using the bedtools suite version 2.3.0 (69). Genes shared between the novel assemblies and their respective reference assemblies were identified by clustering the pooled predicted proteome amino acid sequences with CD-HIT version 4.8.1 (70), using a 70% amino acid identity threshold and a maximum length difference (CD-HIT option -S) of 20 amino acids. Detection of prophage sequences in the reference assemblies was performed using both Phigaro version 2.3.0 (71) and VirSorter2 version 2.2.3 (72). The circular visualizations of the genomic comparisons were generated using the circlize R package version 0.4.14 (73).

Protein sequences for pore-forming toxins were taken from the annotated reference assembly GCF_000013465.1 (strain FPR3757). Signal peptides were identified using SignalP 5.0 (74) and removed. The resulting sequences were identified in assemblies using tBLASTn version 2.12.0 (68), using 99% amino acid identity and 99% query coverage thresholds.

### Mice.

All experiments involving animals were reviewed and approved by the Institutional Animal Care and Use Committee of NYU Langone Health and were performed according to guidelines of the National Institutes of Health (NIH), the Animal Welfare Act, and U.S. Federal Law. Mice were housed in specific-pathogen-free facilities. Female C57BL/6J mice were purchased from Jackson Laboratories at 6 to 8 weeks of age and were randomly assigned to groups of infection. Previously described germfree wild-type (WT) mice were maintained in flexible-film isolators (75), and the absence of fecal bacteria and fungi was confirmed by aerobic culture in BHI, Sabouraud, and nutrient broth (Sigma) and qPCR for bacterial 16S and eukaryotic 18S rRNA genes through sampling of stool from individual cages in each isolator on a monthly basis. Mice were transferred into individually ventilated Tecniplast ISOcages for antibiotic treatment and infection studies to maintain sterility under positive air pressure.

### Growth curve.

To measure the growth curve of S. aureus, cultures grown overnight were subcultured 1:100 into 150 μL of TSB. The diluted cultures were grown for 24 h at 37°C using Bioscreen C. The OD_600_ was measured every 30 min for 24 h.

### Hemolytic activity.

Hemolytic patterns produced by Staphylococcus on sheep blood agar by cross-streaking against RN4220, which produces only beta-hemolysin, were used to semiquantitatively assay hemolytic activity (76, 77).

### Primary human neutrophil killing assay.

Primary human polymorphonuclear leukocytes (PMNs) from anonymous, consenting healthy donors (New York Blood Center) were isolated from buffy coats as previously described (78). Bacterial culture supernatants were prepared by centrifuging (4,000 rpm for 10 min) S. aureus cultures grown in TSB for 5 h and filtering the supernatant through a 0.2 μm filter. PMNs were plated into 96-well flat-bottomed plates at 2.5 × 10^6^ cells per well in medium (RPMI 1640 with 0.1% human serum albumin [HSA] and 0.01 M HEPES). Doubling dilutions of the culture supernatant were added to PMNs and incubated for 1 h at 37°C. After incubation, cell viability was measured using CellTiter (Promega), according to the manufacturer’s instructions.

### Immunoblotting.

Exoproteins of S. aureus filtered culture supernatants were precipitated with 10% (vol/vol) trichloroacetic acid (TCA). The precipitated proteins were washed once with 100% ethanol, air dried, resuspended with 30 μL of SDS-Laemmli buffer, and boiled at 95°C for 10 min. Precipitated exoproteins were separated on 10% SDS-PAGE gels and then transferred to nitrocellulose membranes. The membrane was blocked with 5% milk, probed with the indicated primary antibody, and incubated with Alexa Fluor 680-conjugated goat anti-rabbit or anti-mouse IgG (1:25,000) as a secondary antibody. Primary antibodies used in this study were rabbit anti-LukA (1:5,000) (16), rabbit anti-LukS-PV (1:5,000) (3, 5, 49), rabbit anti-LukD (1:10,000) (42), and rabbit anti-HlgC (1:5,000) (42). Images were acquired with the Odyssey Clx imaging system (Li-Cor Biosciences). Quantification of the band intensity of protein A was performed using ImageJ (NIH).

### Antibiotic conditioning model.

Six-week-old mice were exposed to a cocktail of antibiotics containing metronidazole (1 g/L; Sigma-Aldrich), vancomycin (0.5 g/L; Sigma-Aldrich), ampicillin (1 g/L; Fisher Scientific), neomycin (0.5 g/L; Fisher Scientific), and sucrose (10 g/L) in their drinking water for 13 days. Antibiotics were replaced every 4 days. Mice were given regular water on day 13. Animal weight, water intake, and food intake were measured every 4 days during the treatment period.

### *In vivo* infections.

S. aureus strains and ESKAPE pathogens were grown in TSB and BHI medium, respectively, at 1:100 for 3 h at 37°C with shaking. Bacteria were centrifuged at 4,000 rpm for 5 min and washed two times with phosphate-buffered saline (PBS). The bacteria were OD normalized and diluted to the indicated inoculum in PBS for infection. Mice were anesthetized with Avertin (2,2,2-tribromoethanol dissolved in *tert*-amyl alcohol and diluted to a final concentration of 2.5% [vol/vol] in 0.9% sterile saline) by intraperitoneal (i.p.) injection. For the pneumonia infection model, mice were challenged with 4 × 10^8^ CFU by intranasal instillation of S. aureus. For the bloodstream infection model, mice were challenged with 1 × 10^7^ CFU by retro-orbital injection for S. aureus infections and 5 × 10^7^ CFU for the other ESKAPE pathogens. Signs of morbidity (>30% weight loss, ruffled fur, hunched posture, paralysis, inability to walk, or inability to consume food or water) were monitored after infection.

To evaluate the bacterial burden in organs, infected mice were sacrificed, and the indicated organs were collected in 1 mL PBS and homogenized to enumerate the bacterial burden. Blood was collected by cardiac puncture and anticoagulated using heparin-coated tubes. Hematocrit levels were measured by complete blood counts (CBCs) using a Heska ElementHT5 system.

### Flow cytometry analysis.

To assess the number of neutrophils in lung tissue, single-cell suspensions were prepared as previously described (79). For flow cytometry staining, cells were incubated with Fc block (anti-mouse CD16/32, clone 24G2; Bio X Cell) in fluorescence-activated cell sorter (FACS) buffer (2% fetal bovine serum [FBS] and 0.05% sodium azide in PBS) for 10 min on ice. Cells were stained with peridinin chlorophyll protein (PerCp)/Cy5.5 CD45 (clone 30-F11; BD Biosciences), V500 CD11b (clone M1/70; BD Biosciences), fluorescein isothiocyanate (FITC) Ly6G (clone 1A8; BD Biosciences), BV605 T cell receptor β chain (TCRβ) (clone H57-597; BioLegend), and PacBlue B220 (clone RA3-6B2; BioLegend) for 20 min on ice. Cells were washed twice with FACS buffer and fixed with 200 μL of FACS fixing buffer (2% paraformaldehyde in FACS buffer). Data were acquired using the Cytoflex instrument (Beckman Coulter). Neutrophils were gated as live, single, TCRβ^−^ B220^−^ CD45^+^ Ly6G^+^ CD11b^+^ cells.

### Stool collection and microbial load determination.

Stool samples were collected throughout the 14-day antibiotic treatment period, and DNA was isolated using the DNeasy PowerSoil DNA isolation kit (Qiagen) according to the manufacturer’s instructions. Stool samples were collected throughout the 14-day antibiotic treatment period, and fecal DNA was isolated using the DNeasy PowerSoil DNA isolation kit (Qiagen) according to the manufacturer’s instructions. qPCR for bacterial 16S (UniF340 [5′-ACTCCTACGGGAGGCAGCAGT-3′] and UniR514 [5′-ATTACCGCGGCTGCTGGC-3′]) was performed on a Roche 480II LightCycler and used to determine the microbial load in the intestines of these mice. The relative bacterial burden was calculated using the ΔΔ*C_T_* method, and values were expressed as log fold changes from the values for untreated mice.

### Histology.

Antibiotic-treated mice were infected with a nonlethal dose of 1 × 10^8^ CFU by intranasal instillation, and at 18 h postinfection, the lungs were harvested and placed into 10% neutral buffered formalin (VWR) for 72 h. Tissue was processed by the NYU Experimental Pathology Research Laboratory. Tissues were processed into formalin-fixed paraffin-embedded (FFPE) tissue blocks using a Leica Peloris automated tissue processor. Five-micrometer paraffin sections were stained with hematoxylin and eosin (H&E) on a Leica BondRX autostainer, according to the manufacturer’s instructions. Slides were imaged on a Hamamatsu Nanozoomer whole-slide scanner.

### Induction and assessment of neutropenia.

Cyclophosphamide monohydrate (Sigma) was dissolved in water and injected intraperitoneally at a dose of 150 mg/kg of body weight in a total volume of 200 μL every other day for a week. To assess the number of neutrophils, mice were sacrificed, and cells were isolated from the bone marrow and spleen as previously described (80). Cells were stained for flow cytometry analysis as described above.

### Statistical analysis.

GraphPad Prism 9 was used for all statistical analyses. All of the statistical details of the experiments can be found in the figure legends.

### Data availability.

Raw sequence data and genome assemblies have been submitted to the NCBI and are accessible under BioProject accession number PRJNA831781.
