# Supplementary figures and images for: Microbiome-Independent Effects of Antibiotics in a Murine Model of Nosocomial Infections
Source: mBio. 2022 May 25;13(3):e01240-22. doi: 10.1128/mbio.01240-22 (PMC9239109; doi:10.1128/mbio.01240-22)

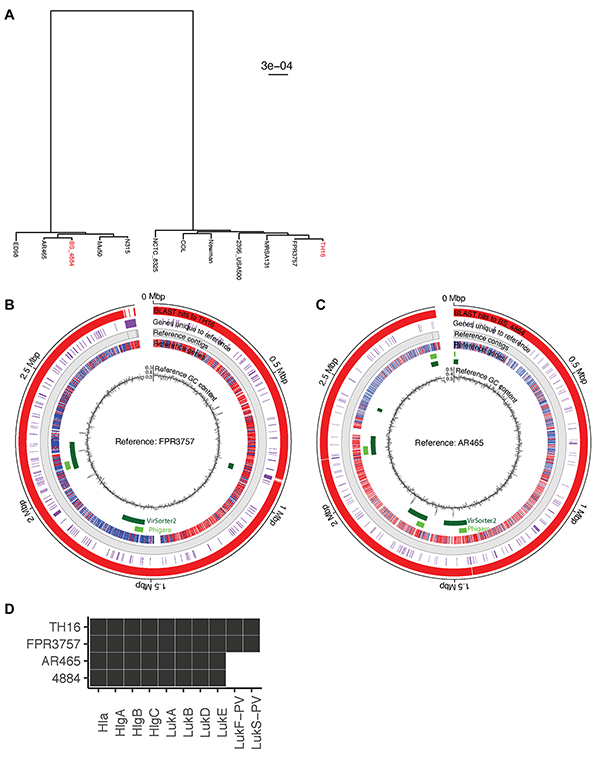

Supplement: FIG S1 [file mbio.01240-22-s0001.tif]

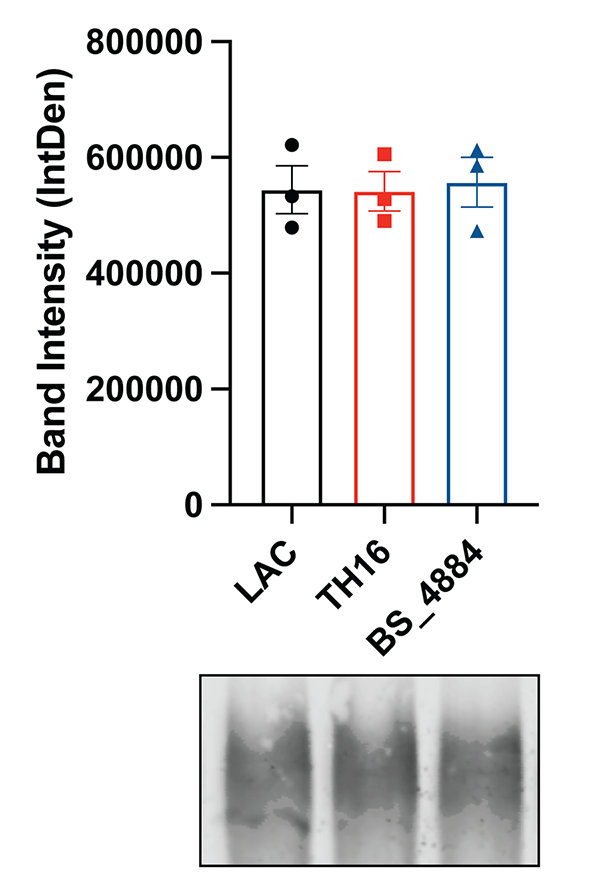

Supplement: FIG S2 [file mbio.01240-22-s0002.tif]

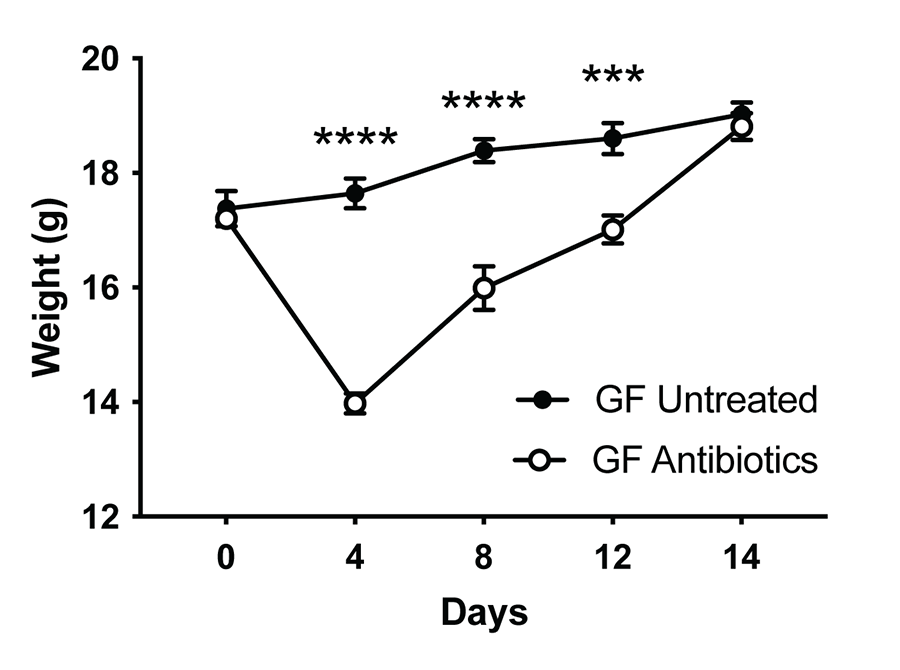

Supplement: FIG S3 [file mbio.01240-22-s0003.tif]
